# Mouse Monoclonal Antibodies Generated from Full Length Human Cereblon: Detection of Cereblon Protein in Patients with Multiple Myeloma

**DOI:** 10.3390/ijms18091999

**Published:** 2017-09-17

**Authors:** Xiubao Chang, Qinqin Xu, Yuexian Hou, Cynthia Li, Ye Xu, A. Keith Stewart

**Affiliations:** 1 Department of Biochemistry & Molecular Biology, College of Medicine, Mayo Clinic Arizona, Scottsdale, AZ 85259, USA; Xu.Qinqin@mayo.edu (Q.X.); hou.yue@mayo.edu (Y.H.); csl95@cornell.edu (C.L.); xuye@bjmu.edu.cn (Y.X.); 2 Division of Hematology, Mayo Clinic Arizona, Scottsdale, AZ 85259, USA

**Keywords:** multiple myeloma (MM), cereblon (CRBN), monoclonal antibody (mAb), epitope, immunohistochemistry (IHC) staining, immunoprecipitation, immunomodulatory drugs (IMiDs)

## Abstract

Immunomodulatory drugs (IMiDs) are profoundly active compounds in the treatment of patients with multiple myeloma (MM). However, despite the fact that treatment with IMiDs has dramatically improved survival for patients with MM, the majority of MM patients develop IMiDs resistance over time. We have found that expression of functional cereblon is required for IMiDs′ action. In addition, it has been reported that cells expressing high levels of cereblon are resistant to proteasome inhibitor, implying that patients with high levels of cereblon should be resistant to proteasome inhibitor. If the above conclusions are correct, cereblon could be considered as a biomarker to determine which standard regimens should be used to treat patients with MM. Unfortunately, the conclusions mentioned above have not been clinically confirmed. In order to confirm these conclusions, we have generated three highly specific mouse monoclonal antibodies (mAbs) against full-length human cereblon. These mAbs can be used to do western blot, immunoprecipitation and immunohistochemistry staining. In addition, their epitopes have been precisely determined and the peptides covering their epitopes completely blocked the antibody binding to cereblon in western blot analysis or in immunohistochemistry staining of MM patients′ specimens.

## 1. Introduction

Multiple myeloma (MM) is generally thought to be incurable, but remissions may be induced with steroids, chemotherapy or stem cell transplants. Immunomodulatory drugs (IMiDs), such as thalidomide, are profoundly active compounds in the treatment of patients with MM [1]. It has been found that the treatment of cancer patients with IMiDs exerts significant effects on immunomodulatory, anti-angiogenic, anti-inflammatory, anti-proliferation and pro-apoptotic activities, etc. [2]. However, despite the fact that treatment with IMiD has dramatically improved survival for patients with MM, the majority of MM patients develop IMiDs resistance over time [3]. Thus, IMiD-resistance is one of the major obstacles for the successful treatment of patients with MM.

What is the molecular mechanism of IMiDs’ action in the treatment of patients with MM? The discovery of cereblon (CRBN) as IMiDs′ target [4] greatly facilitates the investigation of the molecular mechanism of IMiDs’ action. CRBN functions as a recruiter for the well-known E3 ubiquitin-ligase-proteasome-system (UPS) [5,6] and recruits substrates for UPS-mediated degradation. Interestingly, the UPS-mediated protein turnover, via multiple recruiters, regulates virtually all aspects of cellular function. However, the molecular mechanism of CRBN’s action in the regulation of cellular function is largely unknown.

Given the fact that CRBN binds to the cytosolic C-terminus of large-conductance Ca^2+^ activated potassium channel (BK_Ca_) [7,8], the cytosolic C-terminus of a voltage-gated chloride channel-2 (ClC-2) [9], AMP-activated protein kinase (AMPK) [10], proteasome subunit β type-4 (PSMB4) [11], ikaros (IKZF1) and aiolos (IKZF3) [12,13,14,15], homeobox protein MEIS2 [16] and argonaute 2 (AGO2) [17], it is possible that CRBN may recruit these proteins for UPS-mediated degradation. However, it is still not clear why some MM patients are sensitive to IMiDs, whereas others are resistant.

We have found that CRBN expression is required for the anti-myeloma activity of IMiDs [18] and introduction of wild-type or His-tagged CRBN into IMiD-resistant cells increased their sensitivities to IMiD [17]. Recent publications [19,20,21,22,23] also support our conclusion that there may be a correlation between CRBN expression and IMiD sensitivity. However, other publications did not notice the correlation between CRBN expression and IMiD sensitivity in a diverse panel of MM cell lines [24]. Thus, the relationship between CRBN expression and IMiD sensitivity is not fully established.

Interestingly, it has been reported that cells expressing high levels of CRBN are resistant to the proteasome inhibitor induced death [25]. Proteasome inhibitor is widely used to treat patients with MM [26]. Thus, the results mentioned above could be interpreted as that patients with high levels of CRBN should be resistant to proteasome inhibitor treatment. However, this kind of relationship has not been proven clinically. In addition, CRBN has been found to be located in cytoplasm and nucleus [24,25,27]. It has been found that: (1) nuclear CRBN modulates transcriptional activity of ikaros and regulates its downstream target encephalin [28]; (2) mitochondrial CRBN functions as a Lon protease [29]. Thus, clinical value of CRBN measurement to determine the relationship between CRBN expression and IMiD sensitivity or between CRBN expression and proteasome inhibitor sensitivity is a very important issue, whereas determining CRBN subcellular localization is another important issue for finding out CRBN function in each individual cellular organelle. However, it has been reported that most of the commercially available CRBN antibodies are non-specific [24,27].

In this report, we have used full-length human CRBN protein as antigen to generate CRBN-specific monoclonal antibodies (mAbs). Three hybridoma cell lines, which express high levels of CRBN-specific mAbs, were isolated and their epitopes have been determined. These mAbs are highly specific and can be used to do western blot, immunoprecipitation and immunohistochemistry staining.

## 2. Results

### 2.1. 2B11G10, 2F11G5 and 4B9D3 are Highly Specific mAbs for Detecting Human CRBN in Western Blot Analysis

**Figure 1:**
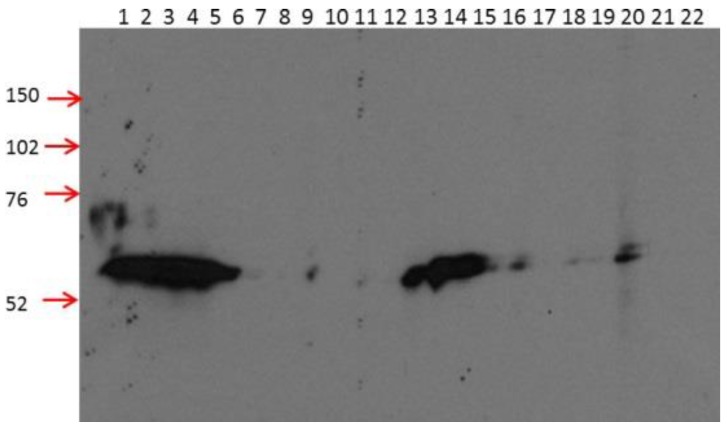
Screening of mAbs generated with full-length human cereblon (CRBN) protein. Whole cell lysates of My5.CRBN [17] were separated on a 7% polyacrylamide gel and probed with the following mAbs generated with full-length human CRBN protein: Lane 1 (L1), the antibody-conditioned media (ACM) of 2B11G10 were diluted 1 fold with 5% milk (in PBST) and L2, undiluted 2B11G10; L3, diluted 2F11G5 and L4, undiluted 2F11G5; L5, diluted 3A2G3 and L6, undiluted 3A2G3; L7, diluted 3A5E8 and L8, undiluted 3A5E8; L9, diluted 3B2B3 and L10, undiluted 3B2B3; L11, diluted 3H3E6 and L12, undiluted 3H3E6; L13, diluted 4B9D3 and L14, undiluted 4B9D3; L15, diluted 4C9F12 and L16, undiluted 4C9F12; L17, diluted 4D12G1 and L18, undiluted 4D12G1; L19, diluted 5A7H10 and L20, undiluted 5A7H10; L21, diluted negative control antibody ACM and L22, undiluted negative control antibody ACM. The numbers on the left side indicate the apparent molecular weight of the markers.

In order to generate mAbs against human CRBN protein, the full length CRBN recombinant protein was used as an antigen to immunize the mice. Hybridoma cell lines derived from the immunized mice were selected and the supernatants derived from these cell lines were used to detect the His-tagged full length CRBN protein expressed in My5.CRBN [17]. As shown in Figure 1, all of these supernatants detected a protein at the expected position (with longer exposure); however, only three of them, i.e., 2B11G10 (#1), 2F11G5 (#2) and 4B9D3 (#7), yielded a strong band at the expected position. In addition, unlike 3B2B3 (#5) or 5A7H10 (#10) which detected multiple bands, including the weak band at the expected position, those three mAbs mentioned above yielded a specific band at the expected position. Thus, these three mAbs are highly specific mAbs for detecting human CRBN in western blot analysis.

### 2.2. 2B11G10, 2F11G5 and 4B9D3 Can Immunoprecipitate Human CRBN from Multiple Myeloma Cell Lysates

**Figure 2:**
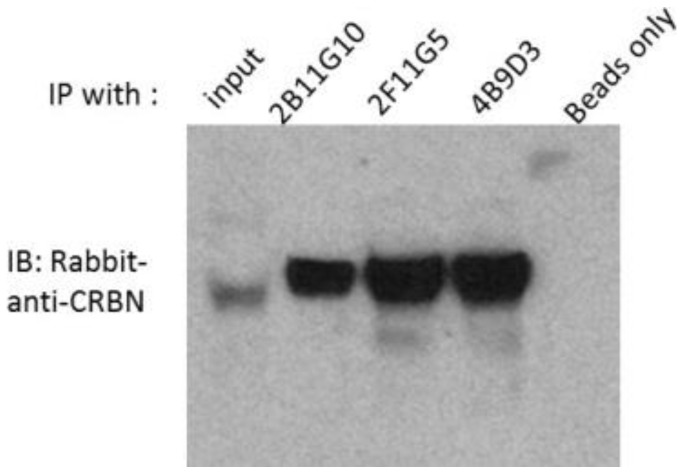
CRBN protein is immunoprecipitated (IP) with mAbs 2B11G10, 2F11G5 or 4B9D3. My5.CRBN cells were lysed with NP40 buffer [17], IPed with the antibodies indicated in the figure and probed with rabbit-anti-CRBN antibody. Input: whole cell lysates; IP: whole cell lysates were immunoprecipitated with antibodies indicated; Control: whole cell lysates IPed with protein G beads without adding primary antibodies; IB: samples probed with rabbit-anti-CRBN antibody.

For the next step, we determined whether these three mAbs can be used to precipitate human CRBN or not. Whole cell lysates of My5.CRBN [17] were used to do the immunoprecipitation. As shown in Figure 2, protein G beads alone did not precipitate human CRBN, whereas 2B11G10, 2F11G5 or 4B9D3 significantly enriched human CRBN protein. In addition, 2F11G5 mAb had proved to be able to co-immunoprecipitate CRBN downstream binding protein argonaute 2 [17].

### 2.3. Determination of Epitopes for 2B11G10, 2F11G5 and 4B9D3

**Figure 3:**
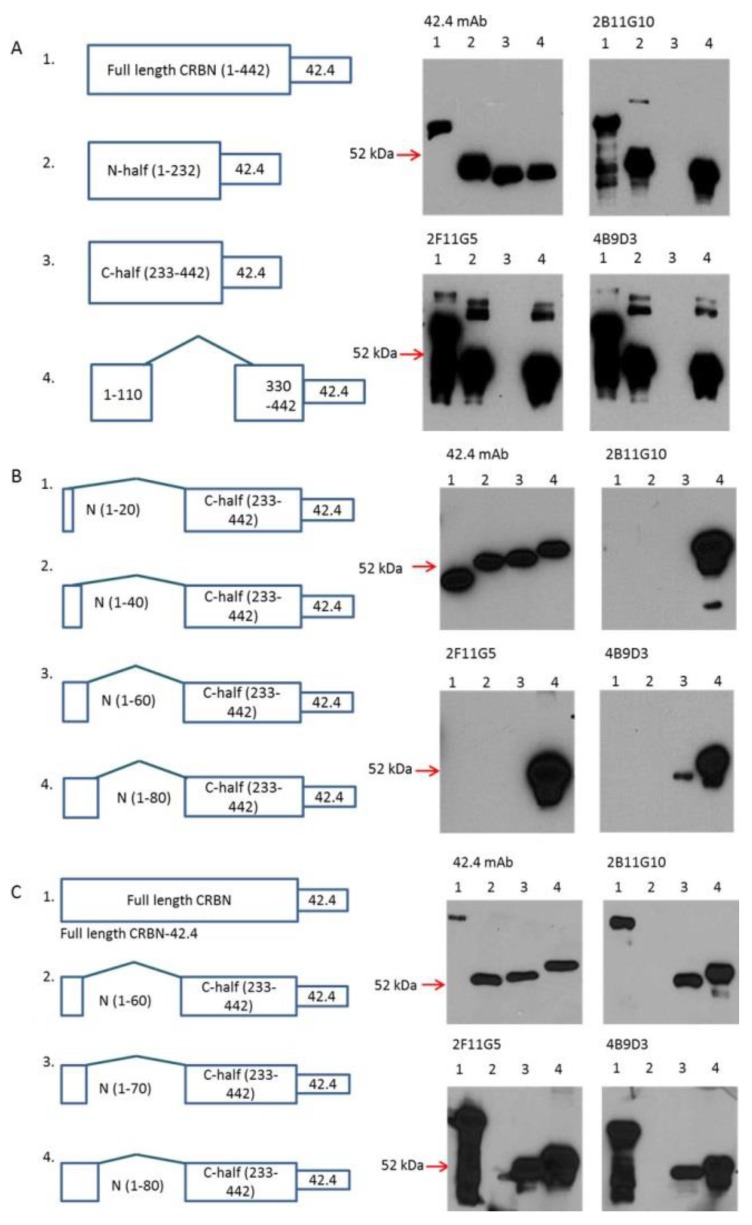
Narrowing down the epitopes of the CRBN mAbs 2B11G10, 2F11G5 and 4B9D3. (**A**) The epitopes of these three CRBN mAbs are located within the *N*-terminal 110 residues. Crude cell lysates of (A1) (MRP1 antibody 42.4 epitope-tagged full-length CRBN (1–442)), (A2) (*N*-terminal half (1–232)), (A3) (*C*-terminal half (233–442)) and (A4) (internal deletion (1–110/330–442)), after sodium dodecyl sulfate polyacrylamide gel electrophoresis (SDS-PAGE), were probed with 42.4, 2B11G10, 2F11G5 or 4B9D3; (**B**) The epitopes of these three CRBN mAbs are located within the *N*-terminal 80 residues. Crude cell lysates of (B1) (MRP1 antibody 42.4 epitope-tagged internal deletion mutated CRBN (1–20/233–442)), (B2) (internal deletion mutated CRBN (1–40/233–442)), (B3) (internal deletion mutated CRBN (1–60/233–442)) and (B4) (internal deletion mutated CRBN (1–80/233–442)] were probed with 42.4, 2B11G10, 2F11G5 or 4B9D3. (**C**) The epitopes of these three CRBN mAbs are located within the peptide of 50–70. Crude cell lysates of (C1) (same as (A1) (1–442)), (C2) (same as (B3) (1–60/233–442)), (C3) (internal deletion mutated CRBN (1–70/233–442)), and (C4) (same as (B4) (1–80/233–442)) were probed with 42.4, 2B11G10, 2F11G5 or 4B9D3.

Since these mAbs were generated from full length CRBN, the locations of their epitopes were not clear. In order to narrow down their epitope location, several CRBN recombinant proteins, including full length, *N*-terminal half (1–232), *C*-terminal half (233–442) and internal deletion between 110 and 330, were expressed in DL21 cells. As shown in Figure 3A, 42.4 mAb [30] detected all 42.4-epitope-tagged CRBN recombinant proteins. In contrast, 2B11G10, 2F11G5 and 4B9D3 mAbs detected full length CRBN, *N*-terminal half and internal deletion mutants, but not the *C*-terminal half recombinant CRBN (Figure 3A), indicating that their epitopes should be located within the peptide from 1 to 110.

To further narrow down their epitopes, several new constructs, including the deletion between 20 and 232; 40 and 232; 60 and 232; and 80 and 232, had been made. As shown in Figure 3B, 42.4 mAb detected all these recombinant proteins. In contrast, 2B11G10, 2F11G5 and 4B9D3 mAbs detected the recombinant protein with internal deletion between 80 and 232, but not the recombinant proteins with internal deletion between 20 and 232; 40 and 232; or 60 and 232; indicating that their epitopes should be located within the peptide of 50–80. To further narrow down their epitopes, a new construct, i.e., the internal deletion between 70 and 232, had been made. As shown in Figure 3C, 42.4 mAb detected all its epitope-tagged recombinant proteins, whereas 2B11G10, 2F11G5 and 4B9D3 mAbs detected full-length CRBN, the recombinant proteins with internal deletion between 70 and 232 or between 80 and 232, but not the recombinant protein with internal deletion between 60 and 232, indicating that their epitopes should be located within the peptide of 50–70.

**Figure 4:**
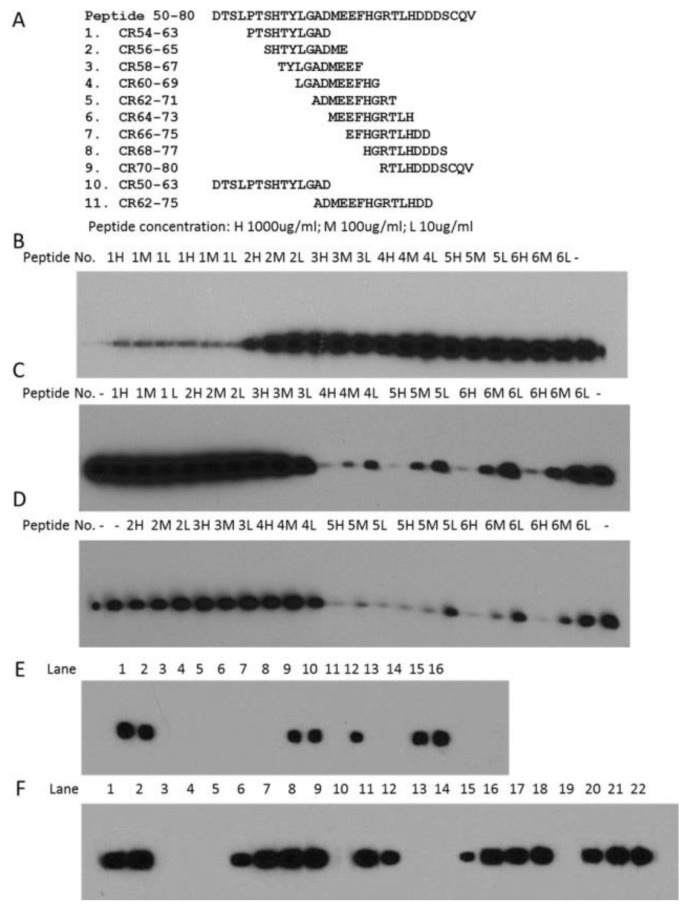
Determination of the epitopes for CRBN mAbs 2B11G10, 2F11G5 and 4B9D3. (**A**) Peptides used to determine the epitopes of CRBN mAbs 2B11G10, 2F11G5 and 4B9D3. The numbers indicate the amino acid positions. For example, CR54–63 indicates CRBN peptide from P54 to D63; (**B**) Peptide CR54–63 partially competed the binding of 2B11G10 mAb to the internal deletion (1–110/330–442) mutated CRBN. Crude cell lysates of the internal deletion mutated CRBN, after SDS-PAGE, were probed with 2B11G10 mAb in the presence of the peptide labeled on top of the gel.—sign indicated that no peptide was added; (**C**) Peptide CR62–71 or CR64–73 partially competed the binding of 2F11G5 mAb to the internal deletion (1–110/330–442) mutated CRBN; (**D**) Peptide CR62–71 or CR64–73 partially competed the binding of 4B9D3 mAb to the internal deletion (1–110/330–442) mutated CRBN; (**E**) Peptide CR50–63 completely competed the binding of 2B11G10 mAb to the internal deletion (1–110/330–442) mutated CRBN. Crude cell lysates of the internal deletion mutated CRBN, after SDS-PAGE, were probed with 2B11G10 mAb in the following conditions: L1–2, 2B11G10 without peptide; L3–4, without 2B11G10; L5–8, 2B11G10 with 1000 µg/mL; 100 µg/mL; 10 or 1 µg/mL of CR50–63; L9–10, 2B11G10 without peptide; L11–12, 2B11G10 with 100 or 10 µg/mL of crude cell lysates of MRP1 antibody 42.4 epitope-tagged *N*-terminal half CRBN (1–232); L13–14, without 2B11G10; L15–16, 2B11G10 without peptide; (**F**) Peptide CR62–75 completely competed the binding of 2F11G5 or 4B9D3 mAb to the internal deletion (1–110/330–442) mutated CRBN. Crude cell lysates, after SDS-PAGE, were probed with 2B11G10 or 4B9D3 mAb in the following conditions: L1–2, 2F11G5 without peptide; L3, without 2F11G5; L4–7, 2F11G5 with 1000 µg/mL; 100 µg/mL; 10 or 1 µg/mL of CR62–75; L8–9, 2F11G5 without peptide; L10–11, 2F11G5 with 100 or 10 µg/mL of crude cell lysates of MRP1 antibody 42.4 epitope-tagged *N*-terminal half CRBN (1–232); L12, 4B9D3 without peptide; L13–16, 4B9D3 with 1000 µg/mL; 100 µg/mL; 10 or 1 µg/mL of CR62–75; L17–18, 4B9D3 without peptide; L19–20, 4B9D3 with 100 or 10 µg/mL of crude cell lysates of MRP1 antibody 42.4 epitope-tagged *N*-terminal half CRBN (1–232); L21–22, 4B9D3 without peptide.

In order to determine the epitopes of these mAbs, peptide ladders, from 50 to 80, had been made (Figure 4A). Preliminary results indicated that peptides CR66–75, CR68–77 or CR70–80 did not have any effect on competition of the antibody binding (data not shown). Thus, these three peptides were not further checked. Our preliminary results also showed that peptide CR54–63 partially competed with 2B11G10 mAb binding, whereas other peptides did not compete at all. To further confirm these results, peptide CR54–63 was checked twice, whereas other peptides were checked only once (Figure 4B). Interestingly, peptide CR54–63 significantly inhibited 2B11G10 mAb binding, but not completely blocked the mAb binding (Figure 4B), suggesting that peptide CR54–63 did not completely cover the epitope of 2B11G10.

Our preliminary results also showed that peptide CR62–71 or CR64–73 partially competed with 2F11G5 mAb binding or 4B9D3 mAb binding, whereas other peptides did not compete at all. To further confirm these results, peptide CR62–71 or CR64–73 was checked twice, whereas other peptides were checked only once (Figure 4C,D). Interestingly, these two peptides significantly inhibited the mAbs binding, but neither peptide CR62–71 nor peptide CR64–73 completely blocked the 2F11G5 mAb binding (Figure 4C) or 4B9D3 mAb binding (Figure 4D), suggesting that neither peptide CR62–71 nor CR64–73 completely covers the epitope of 2F11G5 or 4B9D3.

In order to determine the epitopes of these mAbs, two longer peptides, i.e., CR50–63 and CR62–75, were synthesized (Figure 4A) and used to do the competition for these mAbs binding. As shown in Figure 4E, CR50–63, like the *N*-terminal half CRBN recombinant protein, completely inhibited 2B11G10 binding regardless of whether 1000 µg/mL or 1 µg/mL of the peptide was used, suggesting that CR50–63 completely covers 2B11G10’s epitope. The results in Figure 4F indicated that peptide CR62–75 completely inhibited 2F11G5 or 4B9D3 binding regardless of whether 1000 µg/mL or 100 µg/mL of the peptide was used, suggesting that CR62–75 completely covers 2F11G5 or 4B9D3′s epitope.

### 2.4. Immunohistochemistry (IHC) Staining of Multiple Myeloma Cell Lines with mAb 2F11G5

**Figure 5:**
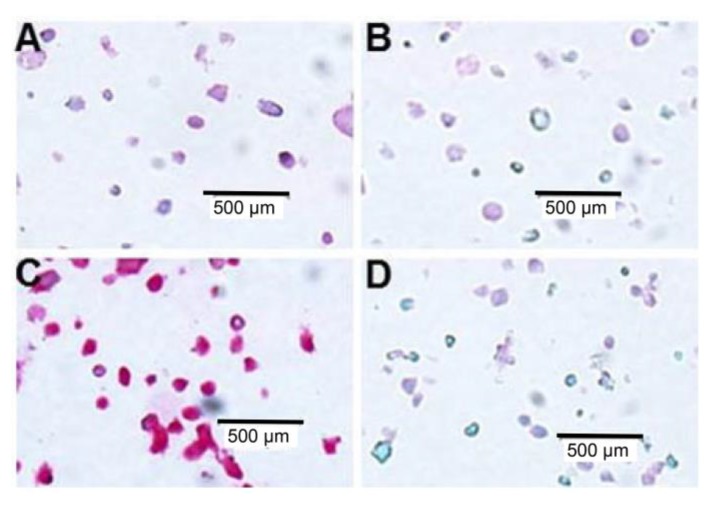
Immunohistochemistry (IHC) staining (Dako’s Permanent Red Substrate) of the CRBN-low and CRBN-high multiple myeloma cell lines. The CRBN-low (MM1.S.Res) cells were stained with CRBN mAb 2F11G5 in the absence of peptide (**A**) or in the presence of CR62–75 peptide covering the mAb’s epitope (**B**) and the CRBN-high (My5.CRBN) cells, in the absence of the peptide (**C**) or in the presence of the peptide (**D**).

We had found that all these three mAbs can be used to stain CRBN in CRBN-expressing cell lines. We then tested whether these mAbs can distinguish the CRBN-high, such as My5.CRBN [17], and CRBN-low, such as MM1.S.Res [17,18], expressing cells or not. In order to do so, the CRBN-high and CRBN-low cells were used to do IHC staining with CRBN mAb 2F11G5. As shown in Figure 5C, 2F11G5 mAb heavily stained My5.CRBN cells, but not the CRBN-low MM1.S.Res cells (Figure 5A). In addition, peptide CR62–75 completely blocked 2F11G5 staining (Figure 5D).

### 2.5. IHC Staining of Multiple Myeloma Patients’ Specimens with mAb 2F11G5

**Figure 6:**
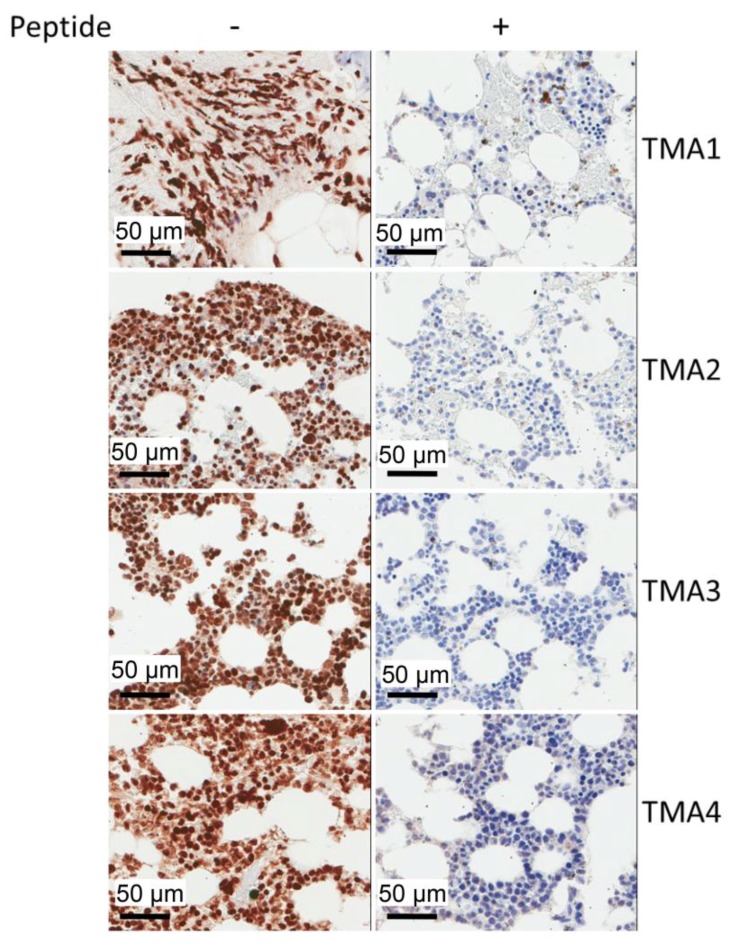
IHC staining (Leica Bond RX stainer) of multiple myeloma patients’ biopsies. Tissue microarray (TMA) of multiple myeloma patients’ specimens (TMA1, TMA2, TMA3 and TMA4) were stained with CRBN mAb 2F11G5 in the absence of the competing peptide CR62–75 (−) or in the presence of the competing peptide CR62–75 (+).

We then tested whether these CRBN mAbs can detect CRBN in paraffin-embedded tissues or not. The results in Figure 6 clearly indicated that the MM cells in patients′ specimens are heavily labeled with CRBN mAb 2F11G5 regardless of which tissue microarray (TMA) is checked. In addition, peptide CR62–75 completely blocked the IHC staining of these tissues, suggesting that 2F11G5 mAb in the supernatant of antibody-conditioned-media is CRBN-specific. Furthermore, nuclei of the MM patients’ cells are heavily labeled with 2F11G5, suggesting that a high percentage of CRBN protein is located in nucleus. Regarding the CRBN protein in cytosol, the localization of CRBN protein in each individual cytoplasmic organelle cannot be determined at the moment and needs to be determined.

## 3. Discussion

Recent publications [17,18,19,20,21,22,23] suggested that CRBN-high cells should be sensitive to IMiD treatment whereas CRBN-low cells should not. However, other publications did not find the tight correlation between CRBN expression and IMiD sensitivity in a diverse panel of MM cell lines [24]. Thus, the relationship between CRBN expression and IMiD sensitivity is not fully established. A recent report indicated that cells expressing high levels of CRBN are resistant to the proteasome inhibitor induced death [25], suggesting that CRBN does play an important role in chemotherapeutic treatment induced cell death. However, the relationship between CRBN expression and sensitivity to proteasome inhibitor has not been clinically established. Thus, clinical evaluation of the relationship between CRBN expression and IMiD sensitivity or between CRBN expression and proteasome inhibitor sensitivity is urgently needed.

In order to evaluate the relationship between CRBN expression and IMiD sensitivity or between CRBN expression and proteasome inhibitor sensitivity, we have generated three mouse mAbs against human CRBN (Figure 1). These mAbs can be used to immunoprecipitate human CRBN (Figure 2). Interestingly, although shorter peptides, such as CR62–71 and CR64–73, can partially block the mAb, such as 2F11G5, binding to the antigen (Figure 4C), only the longer peptide, such as CR62–75, can completely block the antibody binding (Figure 4F), suggesting that these antibodies are highly specific mouse monoclonal antibodies against human CRBN 50–63 peptide (2B11G10’s epitope) or 62–75 peptide (2F11G5 or 4B9D3’s epitope). The results derived from immunohistochemistry staining (Figure 5 and Figure 6) strongly support this conclusion. Furthermore, these mAbs can distinguish CRBN-low cells from CRBN-high cells (Figure 5).

CRBN has been found to be a recruiter for E3 ubiquitin ligase [5,31] and many intracellular factors, including BK_Ca_ [7,8], ClC-2 [9], AMPK [10], PSMB4 [11], IKZF1 and IKZF3 [12,13,14,15], MEIS2 [16], AGO2 [17] and perhaps many other un-identified factors, have been found to be bound with CRBN, suggesting that CRBN regulates the homeostasis of these factors by recruiting them to the UPS-mediated degradation complex. Is this the sole function of CRBN? The results derived from immunohistochemistry staining (Figure 6) indicate that CRBN is located in not only cytosol, but also in nucleus, suggesting that CRBN may have multiple functions. In fact, it has been found that nuclear CRBN modulates transcriptional activity of ikaros and regulates its downstream target encephalin [28], whereas mitochondrial CRBN functions as a Lon protease [29]. Thus, development of highly specific mouse mAbs against human CRBN may provide a tool to determine: (1) the levels of CRBN; (2) the localization of CRBN; (3) the functional roles of CRBN in each individual cellular organelle; and (4) especially to confirm the relationship between CRBN expression and IMiD sensitivity or between CRBN expression and proteasome inhibitor sensitivity in patients with MM.

## 4. Materials and Methods

### 4.1. Multiple Myeloma Cell Lines and Cell Culture

Multiple myeloma cell lines My5.CRBN [17] and MM1.S.Res [18] were used in this project and they were maintained in RPMI-1640 medium (Thermo Scientific, Waltham, MA, USA) supplemented with 10% heat-inactivated fetal bovine serum (Thermo Scientific) and 1% of penicillin (100 I.U./mL) and streptomycin (100 µg/mL).

### 4.2. Generation of Monoclonal Antibodies (mAbs) against Human CRBN

Human full length CRBN recombinant protein, made by GenScript (Piscataway Township, NJ, USA), was used as an antigen to immunize the mice (by GenScript). Subsequent hybridoma cell line screening and subcloning were performed by GenScript. Once they received CRBN antibody-expressing hybridoma cell lines, they collected the antibody-conditioned media supernatants and sent them to us for further tests. Whole cell lysates of My5.CRBN [17] were used for western blot analysis and the cell lines (the supernatants) with higher titles were selected.

### 4.3. Generation of Recombinant CRBN Proteins

CRBN.C-half.FW (Table 1) and CRBN.42.4.RV (Table 1) were used to insert the MRP1 mAb 42.4’s epitope, RENILFGCQL [30], into the C-terminus of CRBN. The 42.4 epitope-tagged full length CRBN cDNA was inserted into the NcoI and NotI cloning sites in pET-32a vector, named as pET-32a.CRBN.42.4.8. This fusion protein should have 158 amino acids from thioredoxin gene (in pET-32a), 442 amino acids from CRBN and 10 amino acids from the 42.4’s epitope. CRBN.N-half.FW and CRBN.N-half.RV; CRBN.C-half.FW and CRBN.C-half.RV; CRBN.110/330.FW and CRBN.110/330.RV; d20/233.FW and d20/233.RV; d40/233.FW and d40/233.RV; d60/233.FW and d60/233.RV; d70/233.FW and d70/233.RV; or d80/233.FW and d80/233.RV were used, pET-32a.CRBN.42.4.8 as template, to make CRBN *N*-terminal half fusion protein (named as pET-32a/N-half (1–232)/42.4); *C*-terminal half fusion protein (pET-32a/C-half (233–442)/42.4); internal deletion between 110 and 330 (pET-32a/CRBN/1-110/330-442/42.4); internal deletion between 20 and 233 (pET-32a/CRBN/1-20/233-442/42.4); internal deletion between 40 and 233 (pET-32a/CRBN/1-40/233-442/42.4); internal deletion between 60 and 233 (pET-32a/CRBN/1-60/233-442/42.4); internal deletion between 70 and 233 (pET-32a/CRBN/1-70/233-442/42.4); internal deletion between 80 and 233 (pET-32a/CRBN/1-80/233-442/42.4) by using QuikChange site-directed mutagenesis kit from Stratagene. After a target plasmid was established in BL21(DE3) and cell density reached 0.6 (OD_600_), the expression of the target protein was induced by the addition of isopropyl β-d-1-thiogalactopyranoside (IPTG) to a final concentration of 0.4 mM. After addition of IPTG, the cells were incubated for another 3 h at 37 °C.

### 4.4. Immunoblotting and Immunoprecipitation

Western blot was performed according to the routine protocol. For CRBN recombinant proteins expressed in BL21(DE3) cells, the cells were lysed with sodium dodecyl sulfate polyacrylamide gel electrophoresis (SDS-PAGE) sample buffer at 70 °C. For CRBN recombinant proteins expressed in multiple myeloma cells, the cells were re-suspended in NP40 cell lysis buffer (0.1% NP40, 150 mM NaCl, 50 mM Tris, 10 mM Sodium Molybdate, pH 7.6), supplemented with 1 × protease inhibitor cocktail (Aprotonin, 2 µg/mL; Benzamide, 121 µg/mL; E64, 3.5 µg/mL; Leupeptin, 1 µg/mL; and Pefabloc, 50 µg/mL) and cells were broken by sonication. Samples were subjected to SDS-PAGE, followed by transferring the proteins to nitrocellulose membranes, probed with primary antibody (indicated in Figure legend) overnight at 4 °C, washed with phosphate buffered saline containing 0.1% Tween-20 (PBST) and then incubated with appropriate horseradish peroxidase-conjugated secondary antibody. Chemiluminescent film detection was performed according to the manufacturer’s recommendation. Rabbit-anti-CRBN antibody was purchased from Sigma (St. Louis, MI, USA).

Immunoprecipitation was performed according to the protocol used in our laboratory [17]. Briefly, My5.CRBN cells were lysed in NP40 cell lysis buffer and the primary antibody, after pre-cleaning with protein G beads (Invitrogen), was added to the cell lysates and gently rotated overnight at 4 °C. Protein G beads were added to the antibody treated cell lysates and washed with NP40 cell lysis buffer three times. The bound proteins were eluted with 45 µL of 2 × SDS-PAGE loading buffer at 90 °C for 10 min.

### 4.5. IHC Staining

For IHC staining of MM cell lines, the pelleted cells were fixed in 10% formalin, re-suspended in melted Histogel at 58 °C and embedded in paraffin, according to the protocol provided by the manufacturer. Slides derived from paraffin-embedded samples were de-paraffinized with xylene, ethanol and water and then boiled in 10 mM sodium citrate buffer (pH 6.0). IHC staining of the treated slides with CRBN mAb 2F11G5 and permanent red substrate-chromogen (from Dako, Carpinteria, CA, USA) was performed according to the protocol provided by the manufacturer.

For IHC staining of MM patients’ specimen embedded in paraffin, the tissue sectioning and IHC staining were performed at the Pathology Research Core (Mayo Clinic, Rochester, MN, USA) using the Leica Bond RX stainer (Leica Microsystems Inc., Buffalo, IL, USA). The tissue slides were dewaxed and retrieved on-line using the following reagents: Bond Dewax (Leica, Buffalo, IL, USA) and Epitope Retrieval 1 (Citrate; Leica, Buffalo, IL, USA). Tissue slides were retrieved for 30 min. Slides were incubated in Protein Block (Dako) for 5 min. The primary CRBN antibody 2F11G5 was diluted to 1:40 in background reducing diluent (Dako) and incubated for 15 min. The CRBN antibody 2F11G5 and blocking peptides (2F11G5 epitope peptide CR62–75 or a blocking peptide control at 500 µg/mL) were incubated for an hour before starting the Bond RX Stainer. The detection system used was Polymer Refine Detection System (Leica, Buffalo, IL, USA). This system includes the hydrogen peroxidase block, secondary antibody polymer, 3,3′-diaminobenzidine (DAB) and Hematoxylin. Once completed, slides were removed from the stainer and rinsed for 5 min in tap water. Slides were dehydrated in increasing concentrations of ethyl alcohol and xylene prior to permanent cover-slipping in xylene based media.

## Figures and Tables

**Table 1:** List of oligonucleotides used to make recombinant CRBN proteins

Number	Name	Oligonucleotide Sequence (from 5′ to 3′)
1	CRBN.42.4.RV	GCGGCCGCTCACAGCTGACATCCAAAAAGGATGTTTTCTCGCAAGCAAAGTATTACTTTG
2	CRBN.N-half.FW	GAAATACCAGAAGAGAAAGTTTCGAGAAAACATCCTTTTTGG
3	CRBN.N-half.RV	CCAAAAAGGATGTTTTCTCGAAACTTTCTCTTCTGGTATTTC
4	CRBN.C-half.FW	GACGACGACGACAAGGCCATGCATTGTGCAAATCTAACTTCA
5	CRBN.C-half.RV	TGAAGTTAGATTTGCACAATGCATGGCCTTGTCGTCGTCGTC
6	CRBN.110/330.FW	CCTCAAGAAGTCAGTATGGTGGAAATAACAACCAAAAATG
7	CRBN.110/330.RV	CATTTTTGGTTGTTATTTCCACCATACTGACTTCTTGAGG
8	d20/233.FW	GGCAACCACCTGCCGCTCCATTGTGCAAATCTAACTTC
9	d20/233.RV	GAAGTTAGATTTGCACAATGGAGCGGCAGGTGGTTGCC
10	d40/233.FW	GACCAGGATAGTAAAGAACATTGTGCAAATCTAACTTC
11	d40/233.RV	GAAGTTAGATTTGCACAATGTTCTTTACTATCCTGGTC
12	d60/233.FW	CATCACATACATACCTACATTGTGCAAATCTAACTTC
13	d60/233.RV	GAAGTTAGATTTGCACAATGTAGGTATGTATGTGATG
14	d70/233.FW	GAAGAATTTCATGGCAGGCATTGTGCAAATCTAACTTC
15	d70/233.RV	GAAGTTAGATTTGCACAATGCCTGCCATGAAATTCTTC
16	d80/233.FW	GACGACAGCTGTCAGGTGCATTGTGCAAATCTAACTTC
17	d80/233.RV	GAAGTTAGATTTGCACAATGCACCTGACAGCTGTCGTC
